# Earlier sexual debut predicts higher (not lower) levels of father care measured across 12 weeks: an experience sampling study

**DOI:** 10.3389/fpsyg.2023.1199735

**Published:** 2023-06-22

**Authors:** Randy Corpuz, Daria A. Kotov, Rylei L. Donovan

**Affiliations:** Department of Psychology, University of Massachusetts Boston, Boston, MA, United States

**Keywords:** fatherhood, sexual debut, life history theory, male development, ESM

## Abstract

Across the lifespan, males negotiate the tradeoff between current and future reproduction. From a life history theory (LHT) perspective, resources invested into earlier reproduction pose a cost to later reproduction. The age of sexual debut is a commonplace measure of sexual maturation. However, in males, thorarche (age of first ejaculation) and years from thorarche to age of first reproduction both represent milestones related to reproductive timing. A fundamental prediction from LHT is that earlier sexual maturation—a “quantity” strategy—predicts decreased levels of care per offspring. In the current study, we test this straightforward relationship looking specifically at a father’s investment of time. In a sample of first-time fathers, we measured the amount of time spent with their 9-to-12-month infants longitudinally using an experience sampling method (ESM)—an ecologically valid method of collecting self-report data on fathers’ use of time Fathers contributed data on their time allocation across a 12-week period. They reported on ages of sexual debut, thorarche, and the years between thorarche and first reproduction (i.e., current age) was calculated. Only age of sexual debut had a relationship with time allocated toward infants. Importantly however, this effect was in a direction opposite of our LHT derived hypothesis. Males with earlier sexual debut spent more time with their infants. Discussion focuses on the potential contributions to this finding and limitations related to small effect size, methods and measurement, and sample demographics.

## Introduction

Life history theory (LHT) provides a theoretical foundation from which to explore variability in parental allocation of resources—such as time—to offspring. Allocating finite resources to growth, survival, and reproduction requires managing tradeoffs across the lifespan. A critical LH tradeoff is that between current and future reproduction. Resources invested into reproducing earlier will come at a cost to those same resources later in life (Charnov and Berrigan, 1993; Stearns, 2000; Roff, 2012). Sexual developmental milestones— thorarche (i.e., first ejaculate), sexual debut, age of first reproduction—and how the timing of these milestones influence downstream parental investment strategies (and are related to one another) has been a central focus of LHT. Despite variability within and between cultures regarding the timing of reproduction (see Kushal et al., 2022), a disproportionate amount of research on the timing of sexual development (e.g., first menstruation- “menarche”) and parental investment (e.g., time in care) has been with females.

Among human males, less is known about the relationship between a father’s own timing of sexual developmental milestones and levels of paternal care as an adult. In general (and across species), males are expected to be the “faster” sex—a consequence of the asymmetries in the costs of reproduction (i.e., male gametes are abundant across the lifespan while the quantity of female ova are heavily constrained; Trivers, 1974). Despite general agreement that human males are relatively more likely to pursue quantity over quality strategies (see Salmon, 2016 for review), there remains considerable variability *within* males as to the levels of care provided to offspring (Corpuz, 2021) that may potentially be accounted for by the timing of one’s sexual development. Contemporary LHT (as applied to humans) positions reproductive strategies along a “slow” to “fast” continuum. Slow strategists are characterized by *increased* parental investment per child. These parents can afford to and invest more resources in each individual offspring (“quality”). On the other hand, parents demonstrating a fast strategy reproduce early and often—*reducing* investment in each individual offspring in favor of investing resources toward a higher number of offspring (“quantity”). These fast strategists are expected to, on average, reach sexual maturation earlier (i.e., female menarche) and invest relatively less in each individual offspring as part of a “quantity over quality” strategy (Del Giudice, 2009; Ellis et al., 2009; Del Giudice and Belsky, 2011; Belsky et al., 2012; de Baca and Ellis, 2017). In fathers, studying the “clustering” of decreased care with earlier sexual development is more nuanced as there is less consensus on which male sexual developmental milestone is most relevant to theory and/or comparable to the commonly utilized age of menarche in females.

### Human fathers

The utility of LHT when considering variability in age of sexual debut is highlighted in recent reviews (see de Baca and Ellis, 2017; Szepsenwol and Simpson, 2019). As mentioned above, sexual debut can sometimes be used as a proxy of an individual’s life history strategy with an earlier sexual debut being indicative of a fast strategy (Sarma et al., 2018; Aronoff and DeCaro, 2019; Brown and Sear, 2021).

As mentioned above, the onset of menarche in females is a conspicuous signal of sexual maturation (Ellis and Essex, 2007). When assessing pubertal timing for males however, first ejaculation (including nocturnal emission/wet-dreams)—known as “thorarche”-is sometimes treated as analogous to onset of female menarche in medical disciplines (Thomas-Cottingham, 2010; Lilienfeld et al., 2014; Diamond et al., 2015). However, this remains contentious in the literature there as well (Chad, 2020). Self-reported age of sexual debut is also used to measure sexual development and maturation among males across disciplines due to the increased retrospective salience of this developmental milestone for males (Cavazos-Rehg et al., 2009). There is little reason to predict that paternal care’s inverse relationship with age of sexual maturation differs across measures—e.g., correlations among thorarche and sexual debut are moderately correlated (Downing and Bellis, 2009).

In humans, there are several classic LH-related studies that demonstrate a relationship between LH strategies and parenting quality-quantity tradeoff. Harsh and/or challenging environments can influence adult investment in children (Draper and Harpending, 1982; Belsky et al., 1991; Ellis and Essex, 2007; Ellis et al., 2009; Meaney and Szyf, 2022; for intergenerational tradeoffs in rodents). Quality of paternal care can be assessed as direct or indirect care (Kleiman and Malcolm, 1981; Boyette et al., 2019) and faster life history trajectories are associated with decreased levels of both (Brown and Sear, 2021). Some evidence of this “early calibration” has also been found in neuroendocrine research on fathers (Corpuz and Bugental, 2020) and their levels of direct paternal care (Sarma et al., 2018). As with other bodies of work, there is no consensus on how to measure the age that a male reaches sexual maturity.

### Overview

In the current study with first-time fathers in the postnatal period, we expand research specific to the relationship between (a) *earlier* sexual maturation and (b) *lower* levels of paternal care. As an exploratory component of this work, we evaluate this relationship using three candidate self-report measures of sexual maturation — age of thorarche, sexual debut, and time between age of thorarche and first reproduction. We expect all three measures of sexual maturation to be positively correlated with one another and for each to predict portions of the variability measured in the paternal investment of time. In moving beyond a single measurement of the timing of sexual maturation, we hope to potentially uncover nuanced relationships among LH-related milestones related to reproductive behavior (i.e., direct paternal care).

## Methods

### Overview and study design

This data is part of a longitudinal study on maternal and paternal postpartum health outcomes. Our research team conducted three home visits scheduled across a one-year period (starting in the third trimester). Data collection (experience sampling method; ESM) on paternal time allocation was initiated at the third home visit (10-month postnatal visit; *M =* 289.85 days*, SD =* 24.95 days) and continued through the following 3 months (see below). In the current study, only data from this period was used. Predictions and *a priori* analyses will focus on fathers—their own sexual development and their father-infant interactions over a 12 weeks period.

All materials and procedures were reviewed and approved by the University’s Institutional Review Board (IRB). During each home visit, consent forms were explained and signed by participants, and fathers subsequently completed a battery of written surveys. During the final visit, fathers were trained on the experience sampling method (ESM) using their personal device.

### Participants

For the current study, *n* = 194 fathers were enrolled at the beginning of data collection and received ESM training.^1^ Fathers were recruited from multiple sources:hospital birthing or community lactation classes (62.7% %), midwife referrals (15.7%), social media ads (13.6%), or community “Baby Basics” class (2.2%). The remaining 6% of the sample did not report a recruitment source. All participants were residing in Southern California (United States) at the time of data collection.

The average age of fathers in this study was *M =* 32.9, *SD* = 5.4, 84.1% of this sample was married to their child’s mother (at intake) and 77.4% of these fathers held at least a college degree. The median income of this sample^2^ was $50,000–$75,000. Fathers self-reported their race/ethnicity as White (70.6%), Latino/Hispanic (12%), Asian American (5.2%), Black/African American (1.7%), Native American (1.3%), multiracial (2.6%), and other (3.9%). All data were collected between 2014 and 2017. No differences were observed in study variables due to marital status (*p* = 0.79), household income (*p* = 0.61), or self-reported ethnicity (*p* = 0.68).

### Materials

#### Sexual maturation

Fathers self-reported (1) their recalled age of thorarche (including nocturnal emission) (“How old were you when you had your first ejaculate”) (this can include a “wet dream”) (2) age of first sexual intercourse (“How old were you when you lost your virginity?”). They were provided with a space to include age in years and months for both items. Using this data, we also computed the (3) time between^3^ age at first reproduction (i.e., current age) and thorarche. Our initial data analysis plan included using all three variables as manifest indicators on a single latent variable labeled “sexual maturation.”

#### Time invested in direct care

As a measure of paternal care, we measured the time that fathers spent interacting with their infant. We employed an Experience Sampling Method (ESM) that used sending/receiving text messages (Csikszentmihalyi and LeFevre, 1989; Hektner et al., 2007). ESM is useful when data related to participant activity is needed immediately and the potential for retrospective bias is high (Alliger and Williams, 1993).

Fathers were told that researchers were interested in how new fathers were spending their time on “non-working” days. Each father was asked to list the days of the week that they have “off from work” during a typical week.^4^ Texting days were scheduled to occur on a non-working day once every 2 weeks. There were six total texting days following the 10-month home visit. Participants were texted at eight (randomly selected with a minimum of 30 min between texts) times between 8:00 am and 6:00 pm on each of these six non-working days. Participants were told that replies messages had to be within 30 min of receipt. They were asked: “*Whom were you actively interacting with at the time you received this text?* A-Alone; B-baby; C-partner; D-relative; E-friend/neighbor/similar; F-other, please specify.^5^” “Actively interacting” was operationalized during home visits as “an exchange between you and anyone listed that involves some sort of communication, which can be verbal, but also through physical contact, eye contact, or engaging in an activity together.” Participants were told that they could select several options at any time. This protocol was designed in accordance with recommendations in an ESM guidebook (Hektner et al., 2007).

We considered a participant fully compliant if — on each individual day, over the course of the six-day ESM campaign — they responded with the requisite minimum of six out of eight (intelligible, i.e., valid characters) replies within 30 min of receipt. The compliance rate of 91% was universally high and similar to rates found in other ESM studies (Csikszentmihalyi, 2011).

## Results

All analyses were run using SPSS (v. 27) and AMOS (v. 27). The longitudinal nature of ESM measures allows for the construction of a latent growth curve model for paternal care across collection periods (Kline, 2015). The unit of measurement for this variable is the proportion of instances that a father reported interacting with his baby over the total number of replies for that day (Beaulieu and Bugental, 2008).

### Latent variable for paternal care

We built a latent growth curve (LGC) model that captured data from all 6 days of sampling. LGC models—using a structural equation framework—model the trajectory of time structured, repeated measures. The technique accounts for the non-independence of scores from the same participant and includes explicit options for specifying the data derived shape of trajectory (Bauer and Curran, 2003). Texting days were modeled as indicators of the intercept and slope of this growth curve model. The mean intercept value for paternal care GCM is *η*0 = 0.62 (*SE = 0.017*, *p* < 0.001) indicated that the *average* reported time fathers were interacting with infants was roughly 60% of the times they were texted as a baseline. The variance for the paternal care GCM was var. (*η*0) = 0.02 (*SE* = 0.006; *p* < 0.001) which indicated substantial variability about this mean level of time that fathers spent with infants at baseline.

The mean slope value for the paternal care GCM is *η*1 = −0.01 (*SE* = 0.026, *p* = 0.65)—indicating that the average change across the measurement period was essentially zero (“flat”). The variance estimates for the paternal care GCM slope factor, var. (*η*1) = 0.03 (*SE* = 0.014, *p* = 0.02), revealed minimal individual variation among the slope across participants. The intercept and slope factors were not related (cov (*η*1, *η*0) = −0.01, *SE* = 0.008, *p* = 0.35). Taken together, we removed the slope growth factor (*η*1) to transform this latent variable into a more parsimonious “intercept-only” model (Bauer and Curran, 2003). All six manifest items (i.e., days) loaded sufficiently onto the remaining latent intercept for paternal care (all *p*s < 0.001).

### Latent variable for sexual maturation

Initially, we attempted to construct a latent variable for “sexual maturation” using the following three indicators: age of thorarche, age of sexual debut, and the amount of time between age at first reproduction (i.e., current age) and thorarche. As an initial step, we constructed a latent variable for sexual maturation using the following three indicators: (1) thorarche (*M* = 12.79, *SD* = 1.65 years); (2) sexual debut (*M* = 17.94, *SD* = 3.38 years); and (3) time between age at first reproduction (i.e., current age) and thorarche (*M* = 20.46, *SD* = 5.64 years).

Thorarche and sexual debut loaded onto latent sexual maturation well (*ps* < 0.01; Table 1). However, in this sample, males who experienced earlier ages of thorarche (*r* = −0.27, *p* < 0.001) and earlier ages of sexual debut (*r* = −0.21, *p* < 0.01) had a *longer* delay between sexual maturation and age of first reproduction (see latent indicator correlations in Table 2).

**Table 1:** Parameter estimates and critical ratios for the initially proposed latent variable “sexual maturation.”

Parameters	Unstd.	SE	CR	*p*	Std.
**Sexual maturation loadings**					
V1	1.00	*nt*	*nt*	*nt*	0.67
V2	1.53	0.57	2.67	***	0.53
V3	−2.08	0.09	−2.70	**	−0.40

“nt” = not tested (parameter fixed to set scale for latent indicators); (V1) thorarche; (V2) sexual debut and (V3) time between age at first reproduction (i.e., current age) and thorarche. ^*^p < 0.05, ^**^p < 0.01, ^***^p < 0.001.

**Table 2:** Correlations, means, and standard deviations for indicators for the initially proposed “sexual maturation.”

	V1	V2	V3
V1	1.00		
V2	0.354***	1.00	
V3	−0.266***	−0.212**	1.00
Mean (age years)	12.79	17.94	20.46
SD	1.65	3.38	5.64

*N* = 194. V = variable. (V1) thorarche; (V2) sexual debut and (V3) time between age at first reproduction (i.e., current age) and thorarche. ^*^p < 0.05, ^**^p < 0.01, ^***^p < 0.001.

**Figure 1:**
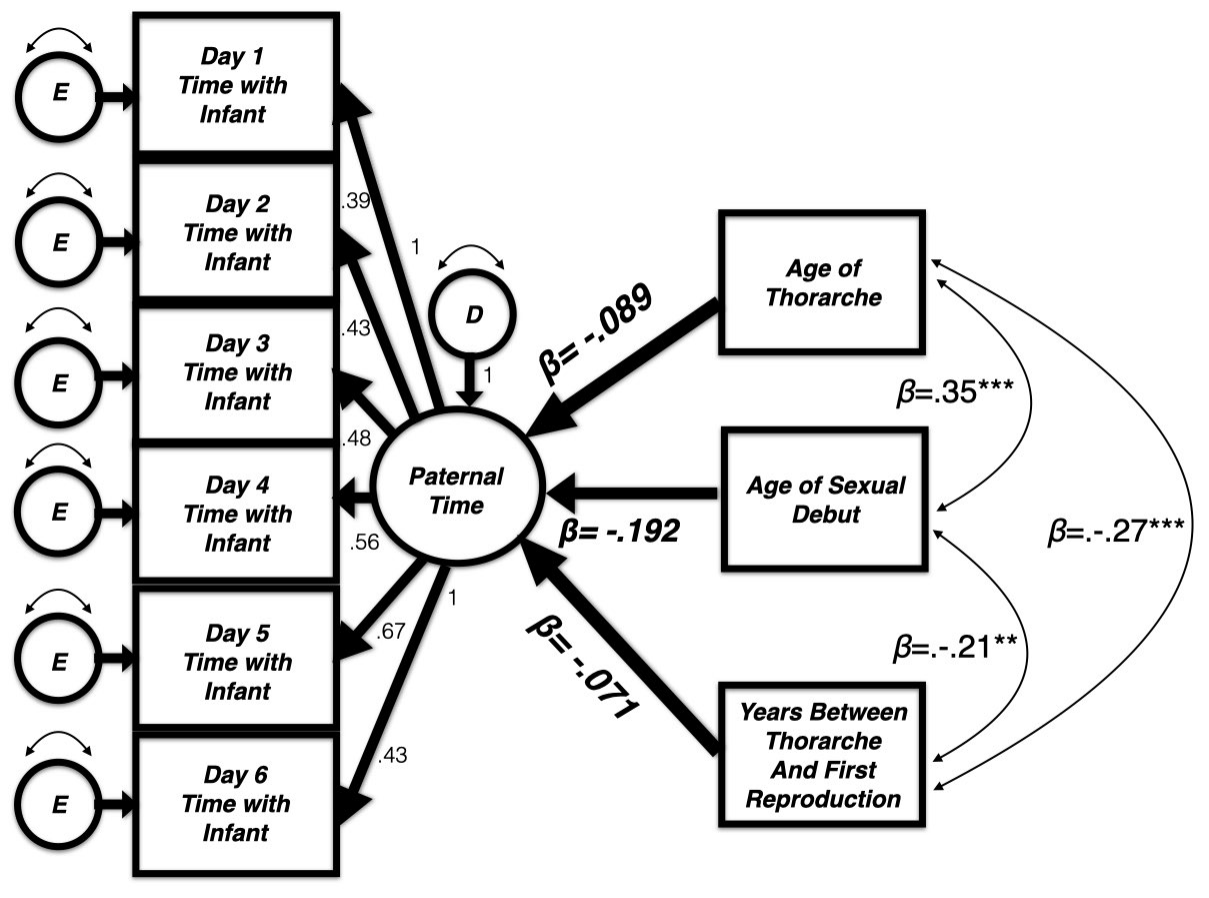
Three measures of sexual maturation (covaried) simultaneously predict the latent variable for time caring for infants as measured using an experience sampling method (ESM). Model fit: [(*χ*^2^(24) = 33.66, *p* = 0.09); CFI = 0.990, RMSEA = 0.049 (90% CI = 0.00–0.08)].

Modeling a latent variable using raw data with an one indicator loading in the opposite direction of two others is permissible. However, interpretation of findings becomes difficult, and the broader implications of this unexpected result would be obscured. As a result, we moved away from creating a latent variable for sexual maturation. We also decided against building three separate models for each indicator and, instead, included all three predictors simultaneously (covarying with one another) to predict the latent variable constructed for paternal care The resulting analysis (Figure 1) more closely resembles our goal of identifying the unique and shared variance in paternal time allocation accounted for by measures of sexual development.

### Covariates

Other variables related to time allocation (as a whole) may be related to a father’s allocation of time during off days across the collection period. While fathers were responding to ESM messages on their self-reported days off, it is possible that the amount of time they spend at work during the week may influence the amount of time they spend with their infants on off days. It is also possible that fathers might spend more (or less) time with infants during their off days depending on how much their partners (mothers) work outside of the house. Using three categories for employment (fulltime, parttime, unemployed), we did not find a relationship between mother employment (*p* = 0.41) nor father employment, (*p* = 0.90) and paternal time allocation on days off.

### *Post-hoc* power analysis

A *post hoc* power analysis of the above model was conducted using Soper (2023) SEM sample size calculator. Using a conservative anticipated effect size (0.10), 0.8 power, one proposed latent variable (paternal care) and three observed variables (sexual development), the recommended sample size was *n* = 200 fathers. The recommended minimum sample size to detect an effect was *n* = 87 at the 0.05 level.

### Hypothesis testing

The paternal time allocation latent variable was extended to add simultaneous (covarying) predictors: (1) age of thorarche or “wet dream” (nocturnal emission); (2) first sexual intercourse; (3) time between age at first reproduction and thorarche. Parameter estimates for this final model appear in Table 3. The final model (Figure 1) utilized in this analysis fits the data adequately [(*χ*^2^(24) = 33.66, *p = 0*.09); CFI = 0.990, RMSEA = 0.049 (90% CI = 0.00–0.08)].

**Table 3:** Parameter estimates for final model.

Predictors of latent paternal care	Unstd.	SE	CR	*p*	Std.
Age of thorarche →	−0.006	0.007	−0.884	0.377	−0.089
Age of sexual debut →	−0.007	0.004	−1.847	0.065	−0.192
Years between thorarche and first reproduction →	−0.001	0.002	−0.74	0.459	−0.071
**Covariance**					
Thorarche <– –> Debut	1.797	0.412	4.367	***	0.351
Years between thorarche and first reproduction <– –> sexual debut	−3.708	1.376	−2.695	**	−0.21
Years between thorarche and first reproduction <– –> thorarche	−2.435	0.714	−3.412	***	−0.266

Measures of sexual maturation simultaneously predicting the intercept-only “paternal care” latent variable. ^*^p < 0.05, ^**^p < 0.01, ^***^p < 0.001.

In this model, (a) fathers’ age of thorarche was not related to the amount of time spent with infants during the 12-week collection period (*β* = −0.09, *p* = 0.38). The same null effect is evident when looking at the (b) the amount of time between a father’s thorarche and first reproduction (*β* = −0.07, *p* = 0.46). However, a father’s age of sexual debut predicted some variance in paternal time allocation. (c) Contrary to our predictions however, fathers who experienced sexual debut at *earlier* ages spent *more time* with their infants (*β* = −0.19, *p* = 0.07). While not statistically significant, this correlation is in a direction opposite of our prediction.

## Discussion

In this U.S. sample of first-time fathers, the results of our analyses revealed a relationship between age of sexual maturation and levels of paternal investment of time. However, this small effect was in the opposite direction of predictions, and only existed for one of our three measures of sexual maturation: age of sexual debut. Fathers who had an earlier sexual debut invested *more* time in care for their infants (measured using ESM). Neither the age of thorarche nor the number of years between thorarche and first reproduction could account for any remaining variability in the amount of time fathers spent with their infants. In addition to this main finding, we found that fathers who experienced early thorarche were indeed younger at sexual debut (putatively “fast” strategists), but these males waited *longer* to produce offspring after thorarche.

### Earlier sexual debut and increased care

Of the three measures of sexual maturation explored in this paper, only sexual debut—perhaps most routinely used among recent LH work—predicting paternal care is unsurprising. However, in this sample, fathers who had earlier ages of sexual debut invested more (not less) in the care of their children. There are a handful of papers on the costs of reproduction and tradeoffs where findings are inconsistent with or inconclusive to other findings on the timing of reproduction (Penn and Smith, 2007; Sear, 2007; Lawson and Borgerhoff Mulder, 2016; Nolin and Ziker, 2016; Gurven et al., 2017). In the current sample, we will speculate on two separate, but complementary contributions to this effect. We reiterate the speculative nature of our discussion; our data cannot fully address the possibilities below but contextualizing our findings in the broader LH literature may be of interest to ongoing work on LH theory as applied to male reproduction.

### Resource abundance

The fathers in this correlational study were, on average: well-nourished, well-developed, visibly healthy, and inhabited WEIRD (Western, Educated, Industrialized, Rich and Democratic; Henrich et al., 2010) environments (see Limitations below). One possibility is that tradeoffs in such plush conditions differ as the cost/benefits of “trading off” are fundamentally different. Van Noordwijk and De Jong (1986) coined the term “big car-big house problem” (see also Sear, 2020). Assumptions underlying LH tradeoffs include that resources in the environment are scarce. In environments where resources are readily available, however, parents face a different tradeoff: diminishing returns on investing in quality over quantity. In high resource environments, parents can “afford” to take more risk and devote resources to fitness in a pattern that differs from that in challenging environments. The risks of buying a “big car” *and* a “big house” are mitigated by the higher quantity of resources available to pursue both.

As one example, Bugental and Beaulieu (2003) and Beaulieu and Bugental (2008) found that parents took greater risks in investing more (not less) in higher-risk (low phenotypic quality) children when parents had high resources—a finding counter to parental investment in most non-humans (see Davis et al., 1999 for computer simulations with birds). As with this work which revealed a layer of nuance to parental investment predictions, it may be the case that strategies tied to earlier sexual debut unfold in a different way than predicted in LH theory when considering the relative costs and risks in higher-resourced environments. Recently, Richardson et al. (2020) found evidence that the relationship between earlier sexual debut and other indicators of a fast LH strategy (e.g., age of first reproduction) differed in higher income individuals (see also Wells et al., 2019) partially supporting the idea that as costs/benefits change, strategies managing reproductive tradeoffs may demonstrate more flexibility.

In the current study, those with earlier sexual debut (putatively, a quantity strategy) pursued the risky strategy of increasing parental care per offspring (quality strategy) under conditions where trading off one for the other is less necessary. It may be that earlier sexual debut (“fast”) males are more sensitive and responsive to changes in resource availability than those males on a “slower” trajectory. The level of care that males with earlier sexual debut invest may be more facultative than males with later sexual debut who’s investment strategies are more stable across one’s reproductive window (i.e., interaction). The idea of revising LH predictions to include additional interacting axes of variation (e.g., an axis on the timing of reproductive events and a separate, related axis of quantity vs. quality tradeoffs) is an important one (Bielby et al., 2007). However, employing this rationale for the current small effect is purely speculation as more research is needed that can identify these proposed interactions.

### Mating effort

Across taxa, separating parental investment and mating effort is difficult as much of parental effort can also be categorized as mating effort (Smuts, 1992). In biparental species, males that provision offspring will also benefit the offspring’s mother’s fitness. Females choose males who are able and willing to invest and, as a result, males advertise their ability to do so. What might appear to be parental investment also influences a male’s ability to attract, retain, and produce additional offspring (and/or reduce interbirth intervals). Across biparental species, males divide efforts uniquely based on resources available to them and the demands (e.g., pathogen load) of the local environment (see Gray and Anderson, 2012). In humans, females often show some preference to mate with males who invest in offspring as has been well documented in research on humans (Buss and Shackelford, 2008; Kaplan and Gangestad, 2015).

In the current study, males who experienced their sexual debut earlier were those who spent more time with their children. While initially counterintuitive, if the observed increase in *parenting* effort is reconceptualized instead as an increase in *mating* effort, the implications of our findings change. Mating effort in much of the human LH literature has become synonymous with the pursuit, acquisition, and retention of mates with an inordinate focus on “short-term” mating, extrapair copulations, (in)stability of pair bonds, quantity of sexual partners, antagonistic romantic relationships (Gladden et al., 2009; Olderbak and Figueredo, 2009). Within this zeitigest, the statement “a male is engaging in high mating effort” may be more likely to elicit images of a male engaged in intra or intersexual competition than a male spending time caring for offspring. One can argue however that, in a biparental species like humans, increases in mating effort (attracting and retaining) in males should look more like the latter than the former. Most human reproduction occurs in the context of long-term relationships (Twenge et al., 2017). Human females can produce offspring in relatively quick succession with levels of paternal support contributing to the length of interbirth intervals (Szabó et al., 2017). Reducing paternal investment in offspring—in any scenario-is a risky wager. Paternal care can have a sizable positive influence on offspring outcomes (see Gray and Anderson, 2012), and present severe detriments to offspring development when wholly absent (see Sigle-Rushton and McLanahan, 2004). If increases in mating effort (“fast” strategy) are adaptive, this increase in mating effort may manifest as behaviors geared toward retaining and eliciting future reproduction with one’s current mate. This would be especially true for fathers in this study with primiparous mothers squarely in their reproductive window with highly neotenous offspring (10–12 months old) that can maximally benefit from paternal care. This line of thought may also be evident in the finding that earlier sexual debut was associated with waiting longer to reproduce. Earlier sexual debut males might be more “sensitive” to environmental conditions and can more readily modulate their chosen mating effort strategy. We speculate that a “fast” strategy—depending on current conditions—might include earlier sexual debut accompanied with investing *more* in offspring to retain a fecund female and/or reduce her interbirth intervals while facilitating the survival of dependent offspring.

Again, we reiterate the speculative nature of this understanding of our findings. However, our results align with recent calls for a re-thinking of LH theory and increased precision of predictions specific to tradeoffs (see Sear, 2020 for expansive advocacy for these improvements).

### Limitations

This study and the current brief report has limitations and we engage with two of these below. Readers are encouraged to interpret our small effect as only an initial result that awaits replication.

As mentioned above, a major limitation to this work is the demographic characteristics of the fathers in this sample. A large portion of fathers in this study were recruited from birthing courses that may already be populated by “slow” strategist fathers demonstrating paternal care ahead of their child’s birth. Fathers were mostly well-educated, White, and married or living with their partner at the time of their infant’s birth. The median income of the fathers in this sample—roughly $69,000 (see Methods)—was well above the median U.S. income recorded during the census immediately preceding data collection ($50,046 in 2010).^6^ The relationship demonstrated in this paper may be constrained to only those families that are healthy, nourished, and in safe, high-resource environments. These “plush” conditions may be misaligned with (or absent from) the human ancestral past (Tooby and Cosmides, 1990). Thus, the finding in this brief report may be due purely to the mismatch between the mechanism responsible for managing tradeoffs and the input characteristics of a wholly novel (historically) resource-rich environment. The use of a homogeneous sample has some positives (Jager et al., 2017) — such as the inclusion of certain environmental variable controls (e.g., nutritional status). However, we urge caution as our findings cannot generalize across populations that are routinely studied in the LH literature (Sear, 2020).

Paternal care can be categorized as direct and indirect care (Kleiman and Malcolm, 1981). In this study, we focused only on direct care and exclusively in the form of time spent with infants. This type of direct care is limited and cannot stand in as representative of direct care more broadly. For example, in our prior work (Corpuz et al., 2021), we found that attenuated levels of testosterone across the first year of an infant’s life predicted spending more time with infants as compared to those with more pronounced increases in testosterone. However, it was these males with more pronounced surges in testosterone who were more invested in their infants during a fear-inducing activity. In both examples, males are contributing direct care but through different behaviors.

Of the amount of time that males invest in daily components of their lives, only a small portion (1/4th on the high end of estimates) across-cultures is devoted to *direct* paternal care (see Gray and Anderson, 2012). In excluding the potential male investment that comes in the form of provisioning, protection, and one’s own status, our findings reveal only a small portion of paternal care behaviors. Our ESM measure of care neglects a facet of paternal investment central to the study of father-child interactions: the degree of the *quality* of care that males demonstrate with their children across development (Geary, 2000). Future designs should integrate measures of direct care (such as ESM), and additional methods that can capture the quality of care that might be unique to fathers (see Cabrera et al., 2018). Additionally, our decision to focus only on days where fathers were off from paid labor limits our ability to consider the nuance of tradeoffs that fathers face in the allocation of time on a typical work day. For example, fathers may adjust their amount of paid labor as a form of paternal investment (Gurven et al., 2009).

In addition to the limitations of how care was measured in this study, the timing of our data collection on paternal time invested (i.e., infancy) represents a limited window on father-child interactions. The father-child relationship may develop and function in substantively different ways across child development with a child’s age being related to the investment of time and financial resources (Flinn, 1992; Anderson et al., 1999; Geary, 2000). Future longitudinal work should consider extending data collection on paternal care across later developmental periods beyond the infant stage.

### Conclusion

In this paper, we present a brief report of a finding that runs counter to our theory-derived prediction. Those first-time fathers in this study who experienced earlier sexual debut spent more (not less) time with their infants across a 12-week period. While not aligned with life history theory predictions specific to reproductive tradeoffs, the small effect in this study is an important contribution to a (relatively) small body of research on: male sexual maturation, measurement of male developmental milestones, and a male’s downstream parenting behavior following first-time fatherhood. The use of ESM to measure paternal care longitudinally is novel and addresses some of the limitations of self-report measures while adding depth to our understanding of the paternal investment of time spent raising highly dependent offspring. In future research, we encourage the use of more diverse samples of fathers and an increased focus on other facets of paternal care such as provisioning. Contributions to parental investment are wide-ranging and findings that reveal a relationship between the timing of sexual debut and parental investment (in either direction) continues to support the utility of a LHT framework while also highlighting the potential need for more nuanced predictions.

## Data availability statement

The raw data supporting the conclusions of this article will be made available by the authors, without undue reservation.

## Ethics statement

The studies involving human participants were reviewed and approved by IRB-University of California Santa Barbara. The patients/participants provided their written informed consent to participate in this study.

## Author contributions

RC collected data, secured NSF funding using the research proposed and subsequently executed for this manuscript. This involved a large scale longitudinal community study where RC recruited, collected data, managed research team/home visitors, analyzed data, and presented original research. RC, DK, and RD analyzed results and contributed equally to the interpretation of results. DK and RD contributed to statistical decisions and execution. RC wrote the introduction and discussion with portions written by DK and RD. All authors contributed to the article and approved the submitted version.

## Funding

RC supported by DGE-1144085 graduate fellowship (NSF) during all data collection. Participant compensation from BCS-1147671 (Bugental).

## Conflict of interest

The authors declare that the research was conducted in the absence of any commercial or financial relationships that could be construed as a potential conflict of interest.

## Publisher’s note

All claims expressed in this article are solely those of the authors and do not necessarily represent those of their affiliated organizations, or those of the publisher, the editors and the reviewers. Any product that may be evaluated in this article, or claim that may be made by its manufacturer, is not guaranteed or endorsed by the publisher.

## Footnotes

^1^Of the 194 trained to use the ESM, 181 supplied complete time allocation data. Participants were coded as complete or incomplete and both groups were compared on all study variables. There were no differences between groups on any demographic variables (all *p*s > 0.66) or variables related to the predictor or outcome in this study (*p*s > 0.71). Overall, missingness was moderate (Little and Rubin, 2019). Models were fitted using full information maximum likelihood (FIML) estimator.

^2^When asked to provide specific detail on annual income (i.e., non-bracketed) at a subsequent data collection visit, the median income of a subsample of participants (*n* = 141) in this study was $69,992. It was not possible to recover this data from the full initial sample.

^3^We did not use “age of first reproduction” on its own as data collected from all males in this study occurred *directly after* their first reproduction (i.e., current age and age of first reproduction would be identical and would not capture the processes we are interested in this study as these relationships would merely measure the influence of paternal age on care).

^4^A small subset (<10%) of fathers required more individualized scheduling of texting days. They self-reported not being employed, having zero days off during a typical week, or reported having “variable” days off during a typical week. These fathers were contacted on a bi-weekly basis (*via* email or phone) to ask which days they anticipated having “more time away from work-related obligations than other days during the upcoming week.”

^5^On each texting day during the study period, 2–3 research assistants would monitor incoming replies from participants in real time to clarify ambiguous replies directly with the participant. The most common issue that arose was participants responding with “other” without specifying what “other” entailed. These issues were resolved in real time and prior to data entry.

^6^https://data.census.gov
